# Seed-to-Seedling Transition in *Pisum sativum* L.: A Transcriptomic Approach

**DOI:** 10.3390/plants11131686

**Published:** 2022-06-25

**Authors:** Galina Smolikova, Ksenia Strygina, Ekaterina Krylova, Aleksander Vikhorev, Tatiana Bilova, Andrej Frolov, Elena Khlestkina, Sergei Medvedev

**Affiliations:** 1 Department of Plant Physiology and Biochemistry, St. Petersburg State University, 199034 St. Petersburg, Russia; k.strygina@spbu.ru (K.S.); e.krylova@vir.nw.ru (E.K.); bilova.tatiana@gmail.com (T.B.); s.medvedev@spbu.ru (S.M.); 2 Postgenomic Studies Laboratory, Federal Research Center N.I. Vavilov All-Russian Institute of Plant Genetic Resources of Russian Academy of Sciences, 190000 St. Petersburg, Russia; khlest@bionet.nsc.ru; 3 Institute of Cytology and Genetics, Siberian Branch of Russian Academy of Sciences, 630090 Novosibirsk, Russia; vikhorev@bionet.nsc.ru; 4 Department of Biochemistry, St. Petersburg State University, 199034 St. Petersburg, Russia; andrej.frolov@ipb-halle.de; 5 Department of Bioorganic Chemistry, Leibniz Institute of Plant Biochemistry, 06120 Halle (Saale), Germany

**Keywords:** desiccation tolerance, gene expression, germination, post-germination, RNA-seq, *Pisum sativum* L., seeds, transcriptome

## Abstract

The seed-to-seedling transition is a crucial step in the plant life cycle. The transition occurs at the end of seed germination and corresponds to the initiation of embryonic root growth. To improve our understanding of how a seed transforms into a seedling, we germinated the *Pisum sativum* L. seeds for 72 h and divided them into samples before and after radicle protrusion. Before radicle protrusion, seeds survived after drying and formed normally developed seedlings upon rehydration. Radicle protrusion increased the moisture content level in seed axes, and the accumulation of ROS first generated in the embryonic root and plumule. The water and oxidative status shift correlated with the desiccation tolerance loss. Then, we compared RNA sequencing-based transcriptomics in the embryonic axes isolated from pea seeds before and after radicle protrusion. We identified 24,184 differentially expressed genes during the transition to the post-germination stage. Among them, 2101 genes showed more prominent expression. They were related to primary and secondary metabolism, photosynthesis, biosynthesis of cell wall components, redox status, and responses to biotic stress. On the other hand, 415 genes showed significantly decreased expression, including the groups related to water deprivation (eight genes) and response to the ABA stimulus (fifteen genes). We assume that the water deprivation group, especially three genes also belonging to ABA stimulus (LTI65, LTP4, and HVA22E), may be crucial for the desiccation tolerance loss during a metabolic switch from seed to seedling. The latter is also accompanied by the suppression of ABA-related transcription factors ABI3, ABI4, and ABI5. Among them, HVA22E, ABI4, and ABI5 were highly conservative in functional domains and showed homologous sequences in different drought-tolerant species. These findings elaborate on the critical biochemical pathways and genes regulating seed-to-seedling transition.

## 1. Introduction

The transition from a growth-arrested seed to a germinating seed and then to a seedling is a crucial step in the plant life history [1,2,3,4]. During and after germination, early seedling growth is supported by the catabolism of stored reserves of protein, oil, or starch accumulated during seed maturation [5,6]. These reserves support cell expansion and seedling development. Successful seed germination and establishing a normal seedling are determining features in plant propagation, having both economic and environmental importance.

During late maturation, orthodox seeds lose up to 95% of their water and successfully enter dormancy. This feature allows them to sustain unfavorable environmental conditions, such as extremely high or low temperatures and drought [7,8,9,10]. Desiccation-tolerant seeds survive drying to 0.1 g H_2_O/g dry weight or less [2]. The mechanisms behind desiccation tolerance are activated at the late seed-maturation stage and associated with the accumulating LEA proteins, small heat shock proteins, non-reducing oligosaccharides, and antioxidants [9,11,12,13,14]. Regulation of seed maturation and the onset of desiccation tolerance is based on the ABA/GA ratio along with the network of four master regulators, usually referred to as LAFL, i.e., LEAFY COTYLEDON1 (LEC1), ABSCISIC ACID INSENSITIVE3 (ABI3), FUSCA3 (FUS3), and LEC2. Namely, LAFL is directly involved in the coordination of seed maturation and inhibition of seed germination [15,16]. Epigenetic modifications, such as changes in DNA methylation and histone post-translational modifications, are also crucial for seed desiccation tolerance and longevity [17,18,19,20]. The switch of the developmental program from maturation to germination is accompanied by the suppression of LAFL genes and activation of those involved in vegetative growth [16].

The process of seed germination *sensu stricto* can be divided into two phases [2,21]. Phase I is characterized by rapid water uptake, enhanced hydration of macromolecules, activation of respiration, and repair of membranes, mitochondria, and DNA. Phase II is characterized by mobilization of reserves, translation of stored mRNAs, transcription and translation of newly synthesized mRNAs, and activation of protein biosynthesis [22].

Protrusion of a radicle is regarded as the beginning of phase III, which is a post-germination stage. At this moment, germination ends, and the post-germination phase III begins [2]. The transition from germination to the post-germination stage corresponds to the loss of desiccation tolerance [23,24,25,26]. Up to this point, the seeds can lose water without any viability loss, and their metabolic processes can resume upon subsequent rehydration [27,28,29]. This stage, when reversible dehydration/rehydration cycles are still possible without losses of germination efficiency, is often referred to as the “window of desiccation tolerance” [28]. A loss of desiccation tolerance in seeds during germination supposedly occurs upon the resumption of DNA synthesis in the radicle cells with the entry of radicle cells into the G_2_ phase of the cell cycle, during which the cell prepares itself for mitosis [30]. Cells in the G_2_ phase of the cell cycle, with duplicated DNA, were more sensitive to stresses (desiccation in particular) than cells that were still in the pre-synthetic phase G_1_ [31]. Whether seeds maintain dormancy or germinate depends on the balance of phytohormones and environmental factors, such as temperature, moisture, and light [32,33,34,35,36,37]. Seed germination also includes such critical processes as silencing of seed development genes and activation of vegetative growth genes [16,38,39]. Key modulators of the transition from seeds to seedlings are epigenetic modifications such as DNA methylation, demethylation, histone modifications, and sRNA pathways [40,41,42]. Apparently, at this moment, the program controlled by the LAFL network is blocked [43,44]. For example, the LAFL genes are repressed during seed germination by the Polycomb machinery via the histone H3K27 trimethyltransferase activity of the PRC2 and the histone H2A E3 monoubiquitin ligase activity of the PRC1 [38,45,46,47].

To provide novel insights into the mechanisms of the seed-to-seedling transition, we performed transcriptome profiling of embryonic axes isolated from germinated pea (*Pisum sativum* L.) seeds before and after the beginning of radicle growth. Pea is a widespread crop plant representing one of Europe’s global food protein sources. Pea genome was fully annotated only in 2019 [48]. This reference sequence is a valuable tool for high-quality transcriptomic analysis to understand the molecular basis of agronomically important traits and support crop improvement.

## 2. Results

### 2.1. Physiological Status of Germinated Pea Seeds

**Figure 1:**
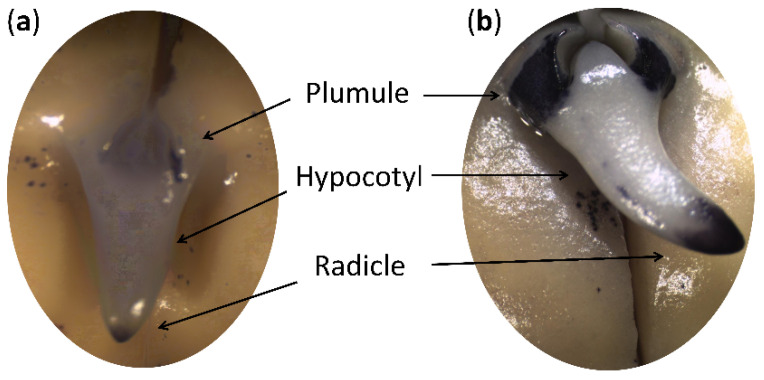
Axes of germinating pea seeds before radicle protrusion (**a**) and after radicle protrusion (**b**). Seeds incubated for germination for 72 h were stained with nitroblue tetrazolium. Dark-blue staining shows the superoxide anion radical localization.

We incubated the pea seeds between moist filter paper layers at 22 °C for 72 h, isolated the cotyledons and embryonic, axes and measured their moisture content. The start of root cell elongation and division, accompanied by the so-called “radicle protrusion,” was observed in 72 h of seed germination. Since that process was not synchronous, we divided the 72-hour-germinated seeds into two batches: (a) before the start of embryonic root growth (before radicle protrusion) and (b) after the start of embryonic root growth (after radicle protrusion). The appearance of axes is shown in Figure 1.

**Figure 2:**
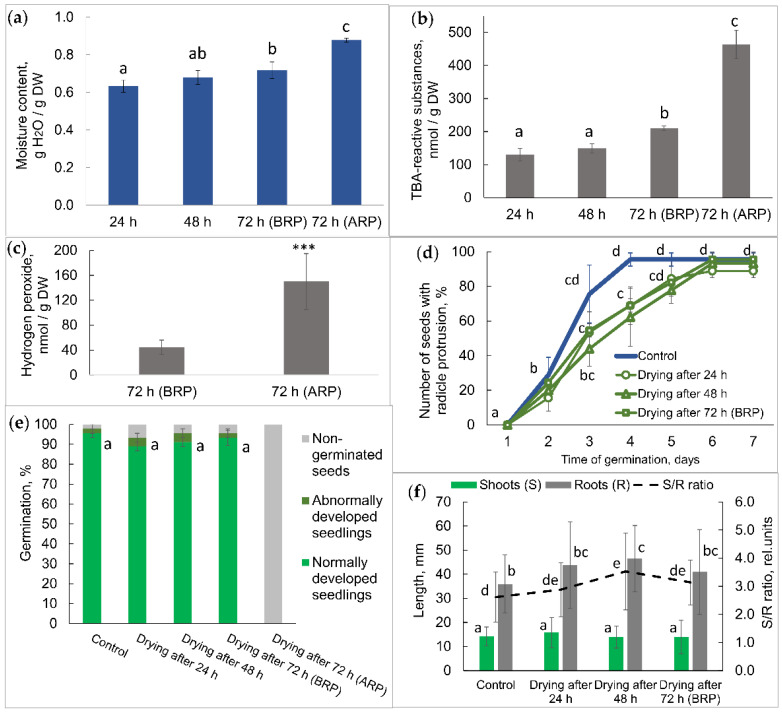
Physiological characterization of pea seeds and seedlings: Control, untreated seeds; BRP, before radicle protrusion; ARP, after radicle protrusion. (**a**) Moisture content in embryonic axes isolated from seeds after 24, 48, and 72 h of germination. The data represent the mean ± standard error of five biological replicates. The statistical significance was determined by the Kruskal–Wallis test. (**b**) Germination rate was recorded as the number of seeds with visible radicle protrusion. The data represent the mean ± standard error of three biological replicates. The statistical significance was determined by the *t*-test. (**c**) Content of thiobarbituric acid (TBA)-reactive substances in seed axes. The data represent the mean ± standard deviation of five biological replicates. The statistical significance was determined by the Kruskal–Wallis test. (**d**) Numbers of normally developed seedlings, abnormally developed seedlings, and non-germinated seeds after nine days of germination. The data represent the mean ± standard error of three biological replicates. The statistical significance was determined by the *t*-test. (**e**) Content of hydrogen peroxide in seed axes. The data represent the mean ± standard deviation of nine biological replicates. The statistical significance was determined by the *U*-test. (**f**) Length of the shoot and root of seedlings after nine days of germination. The data represent the mean ± standard deviation of 14–19 biological replicates. The statistical significance was determined by the Kruskal–Wallis test. Letters above the bars indicate significant differences between the mean values for (**a**,**b**,**d**–**f**) (*p* ≤ 0.05). Asterisks indicate significant differences between the mean values for (**c**) (*p* ≤ 0.001).

Moisture content in the isolated axes before and after radicle protrusion was 72% and 87%, respectively (Figure 2a). Meanwhile, the moisture content in the isolated cotyledons was 65% in both batches. Radicle protrusion was accompanied by ROS generation in seed axes. We observed the accumulation of superoxide anion radical in the embryonic root and plumule (Figure 1). On the biochemical level, the radicle protrusion manifested in an approx. 2-fold increase (*p* ≤ 0.05) in the thiobarbituric acid-reactive substances, expressed as malondialdehyde equivalents (Figure 2b). It was accompanied by a 3-fold increase (*p* ≤ 0.001) in hydrogen peroxide (Figure 2c).

To measure the level of desiccation tolerance in seeds imbibed for 24, 48, and 72 h, they were dried at 22 °C for 24 h to the initial moisture content. It is essential that drying was done only for the seed batch before radicle protrusion. After drying, the seeds were returned to moist filter paper to germinate. Radicle protrusion started after two days of germination and reached 50–60% after three days, as in control and previously dried seeds (Figure 2d). However, in nine days, the total amount of seeds did not statistically differ in all versions. We evaluated the germination after nine days; the assessment covered normally developed seedlings, abnormally developed seedlings, and non-germinated seeds (Figure 2e). Normally developed seedlings had a well-formed embryonic axis, including the root, hypocotyl, epicotyl, and plumule with true leaves (Figure S1). Abnormally developed seedlings had an underdeveloped epicotyl or a too short main root. No statistically significant differences existed between the shoot and root lengths in seedlings grown from the control seeds or previously dried seeds (Figure 2f and Figure S2). It is important to note that drying at the different germination stages up to the visible radicle protrusion did not entail seed damage. However, when we dried seeds after the radicle protrusion, they lost their viability (Figure 2e).

### 2.2. Comparative Transcriptome Profiling of Pea Seed Axes during Seed-to-Seedling Transition

To dissect the molecular basis for massive changes during the seed-to-seedling transition, we performed RNA sequencing of isolated embryonic axes before and after radicle protrusion with three biological replicates. The analysis was performed using the Illumina NovaSeq 6000 SP high-throughput whole-genome sequencing system. After removing the low-quality sequencing reads, a total of 511,182,576 reads were obtained (length of reads: 100 bp on one side of the fragments), with an average of 85 million filtered reads for each replicate.

Quality control of the sequencing results showed the high quality of the reads, with a small number of adapter sequences. After filtering the reads by length and quality and upon removing the adapter sequences, the reads were mapped to the reference Pea Genome International Consortium, version 1a (*Pisum sativum* v1a) [48]. Differentially expressed genes (DEG) with a false-discovery rate (FDR) < 0.05 and log base 2-transformed fold change (|logFC|) > 2 were acknowledged as differentially expressed.

**Figure 3:**
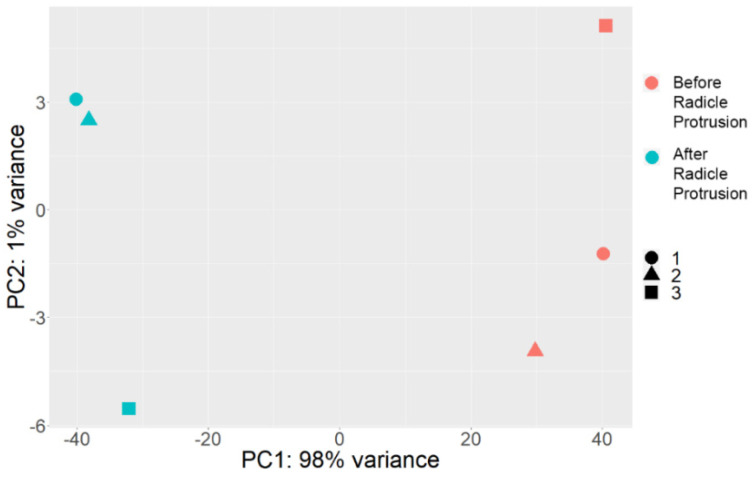
Principal component analysis (PCA) plot of all expressed genes in the RNA−seq data. The *X*−axis indicates the first principal component; the *Y*−axis indicates the second principal component. The percentage of variance explained by each PC is shown in each case. 1–3, biological replicates.

The principal component analysis (PCA) based on the expression of 24,184 genes demonstrated a high level of differences between the seed axes before and after radicle protrusion (Figure 3). The percentage of the explained dispersion for the PCA model was 99% (98% for PC1 and 1% for PC2).

**Figure 4:**
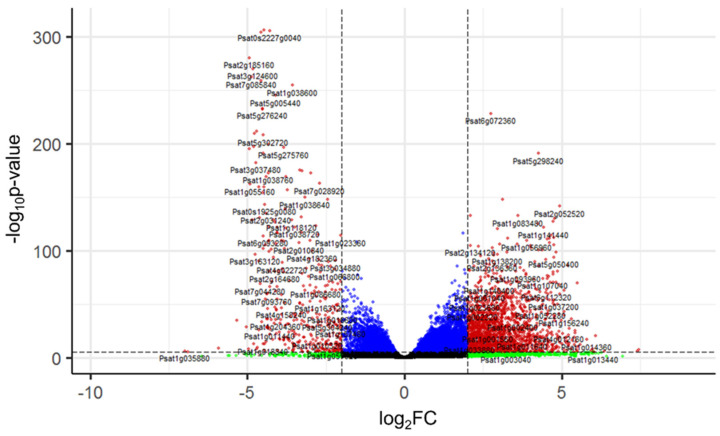
Volcano plot representing 24,184 differentially expressed genes. The *X*−axis indicates the log2−transformed gene expression fold changes between seed axes before and after radicle protrusion. The *Y*−axis indicates the log10−transformed *p*−value. Dashed lines indicate logFC and *p*−value thresholds. The scattered points represent each gene. Significant differentially expressed genes are highlighted in red. Genes with a significant logFC value but nonsignificant *p*-value are highlighted in green. Genes with a significant *p*−value but nonsignificant logFC value are highlighted in blue. The absence of significant differences is highlighted in black.

A volcano plot shows the distribution of differentially expressed genes (DEGs) by FDR and *p*-value. Among them, we observed a 4-fold increase in the expression of 2101 genes and a 4-fold decrease in the expression of 415 genes (|logFC| > 2). Genes with significant expression changes are marked in the diagram with red dots (Figure 4).

### 2.3. Categorization and Functional Annotation of the DEGs in Pea Seed Axes during Seed-to-Seedling Transition

#### 2.3.1. GO Functional Enrichment Analysis

The DEGs specifically identified in seed axes after 72 h of germination were used to map the gene ontology (GO) database to explore significantly enriched terms compared with the genome background using the AgriGO v2.0 toolkit with false-discovery rate (FDR values < 0.05) as the threshold. The number of significant annotated genes whose expression decreased was 250, while the expression of 1361 genes increased. Treemap visualization of the GO terms significantly enriched in downregulated and upregulated genes is shown in Figure S3a,b. We identified the 50 significant GO terms among the downregulated DEGs and 205 significant GO terms among the upregulated ones.

**Figure 5:**
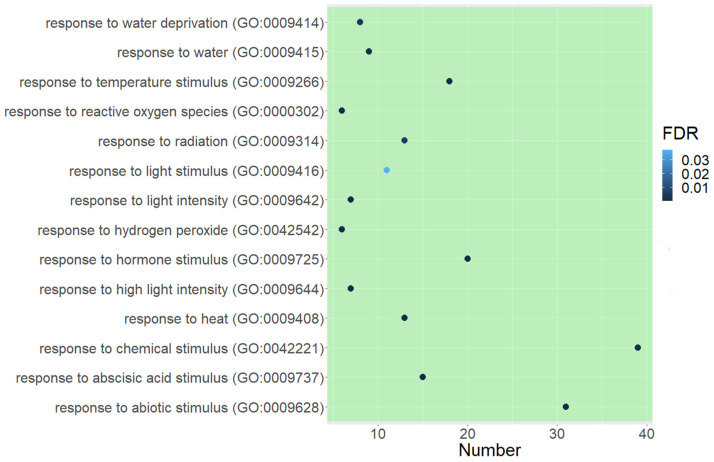
Bubble plot of GO terms associated with the response to stimuli and significantly enriched in genes downregulated in pea seed axes after seed radicle protrusion. The *X*-axis corresponds to the number of genes related to the GO term. The *Y*-axis corresponds to the GO term. The point color indicates FDR: the darker the color, the more significantly enriched the GO term.

In the downregulated gene group, we identified the main categories contributing to the seed development process (“seed development”, “developmental process”, “reproduction”, “multicellular organismal process”, and “regulation of transcription, DNA template”) and seed tolerance (“response to heat” and “response to stimuli”) (Figure S3a). Among them, “response to heat” was the largest group, containing 14 GO terms, including “response to abiotic stimulus”, “response to temperature stimulus”, “response to ROS”, “response to water”, and “response to water deprivation” (Figure 5).

In the upregulated gene group, we identified the large categories containing multiple GO terms: “regulation of biological activity”, “photosynthesis”, “post-embryonic morphogenesis”, and “lipid localization” contributing to the post-germination seedling development (Figure S3b). Four categories belonged to stress tolerance: “response to chitin”, “response to stimuli”, “immune system process”, and “immune effector process”. Three categories belonged to secondary metabolism: “secondary metabolic process”, “plant-type secondary cell wall biogenesis”, and “flavonoid metabolic process”.

#### 2.3.2. Overview of Metabolism

**Figure 6:**
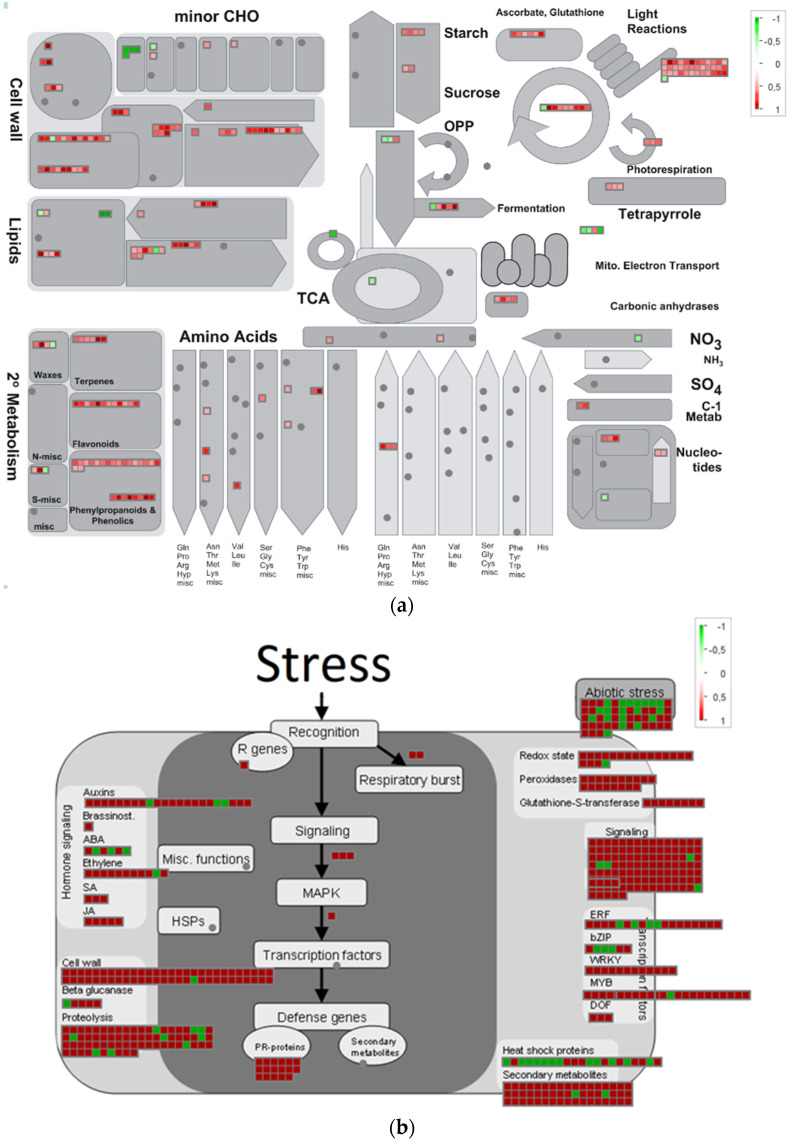
Overview of the changes in the metabolism of pea seed axes after radicle protrusion, obtained using the MapMan software. All squares represent genes with a significant differential expression assigned to the various metabolic pathways. The red color indicates an increase in gene expression; the green color indicates a decrease in gene expression. (**a**) Snapshot of modulated genes over the central metabolic pathways. (**b**) Snapshot of modulated genes over the stress metabolism.

We annotated metabolic pathways using the MapMan software and visualized the changes in the metabolism of pea seed axes after radicle protrusion (Figure 6). As shown in Figure 6a, the seed-to-seedling transition was related to the biosynthesis of cell wall components; secondary metabolism; photosynthesis; metabolism of lipids, sugars, and amino acids; and redox status. Among the cell wall, lipids, and amino acids, some genes were upregulated and some downregulated. Starch and sucrose metabolism, light reaction, and photorespiration were enhanced. Secondary metabolism contained many upregulated genes participating in synthesizing the terpenes, flavonoids, phenylpropanoids, and phenolics.

DEGs were also mapped to genes associated with response to stressors (Figure 6b). Genes related to hormone signaling changed differently. Some genes associated with abscisic acid (ABA) were upregulated, and some were downregulated. At the same time, we observed the increased expression of most of the genes associated with auxin, ethylene, salicylic acid (SA), and jasmonic acid (JA). We divided the abiotic stress genes into equal upregulated and downregulated groups. However, the expression of heat shock proteins was mainly downregulated. The changes were associated with the expression of genes responsible for the cell redox status (ascorbate, glutathione, thioredoxin, and peroxidases). We also identified the upregulation of secondary metabolism genes and genes related to pathogen attack (PR-proteins).

### 2.4. Quantitative PCR Analysis of Selected Genes Related to Water Deprivation

qRT-PCR was performed for sixteen genes to validate the differential expression of genes selected by RNA-Seq. Among them, the seven upregulated DEGs and nine downregulated DEGs were detected in the pea seed axes before and after radicle protrusion (Figure S4a). The results showed that the expression pattern obtained by qRT-PCR corroborated the one obtained from RNASeq data for all sixteen genes. Spearman’s coefficient attested to a significant correlation (R = 0.978), supporting the reliability of sequencing results (Figure S4b).

The eight and fifteen identified DEGs downregulated in seed axes after radicle protrusion were related to GO terms “response to water deprivation” and “response to abscisic acid stimulus”, respectively (Figure S3, Table S6). The DEGs were first annotated with BLASTX search against *A. thaliana* and then mapped to the Pea Genome Assembly v1a.

In the “response to water deprivation” group presumably associated with the loss of drought tolerance by seeds there were the *ABI4 (ABA INSENSITIVE 4*, Psat2g031240), *ABI5 (ABA INSENSITIVE 5*, Psat3g033680), three copies of *LEA14 (LATE EMBRYOGENESIS ABUNDANT 14*, Psat4g157880, Psat4g157760, Psat3g105280), *HVA22 (HORDEUM VULGARE ABUNDANT 22E*, Psat5g052360), *LTI65 (LOW-TEMPERATURE-INDUCED 65*, Psat0s2227g0040), three copies of *RD22 (RESPONSIVE TO DESSICATION 22*, Psat6g033920, Psat6g033960, Psat0s4403g0040), *LTP4 (LIPID TRANSFER PROTEIN 4*, Psat7g227120), and *PER1 (1-CYS PEROXIREDOXIN*, Psat7g085840).

**Figure 7:**
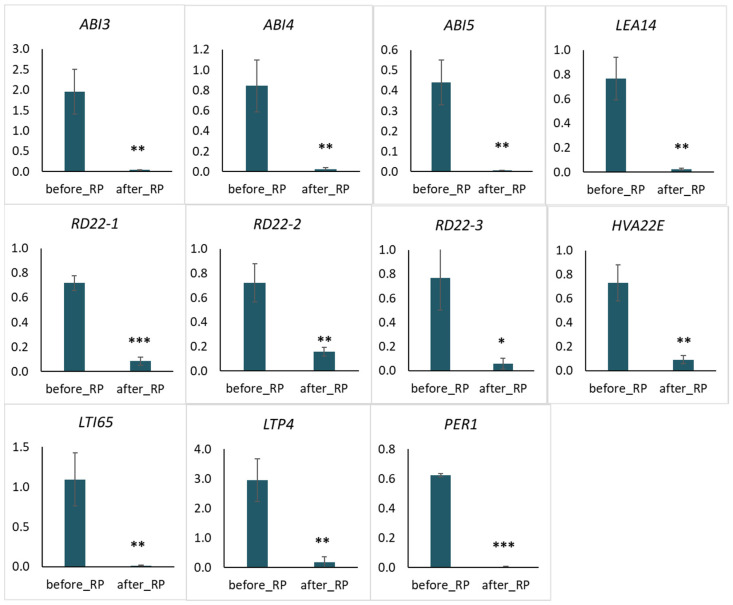
Relative expression levels of the protein-encoding genes ABA INSENSITIVE 3/4/5 (ABI3, ABI4 and ABI5), LATE EMBRYOGENESIS ABUNDANT 14 (LEA14), RESPONSIVE TO DEHYDRATION 22 (RD22), HORDEUM VULGARE ABUNDANT 22E (HVA22E), LOW-TEMPERATURE INDUCED 65 (LTI65), LIPID TRANSFER PROTEIN 4 (LTP4), and 1-CYSTEINE PEROXIREDOXIN (PER1) in pea seed axes before and after radicle protrusion (RP). Y-axis corresponds to relative expression levels analyzed by qRT-PCR. Data were normalized to the expression of the Psat1g039040 (GenBank Z25888) gene encoding the phosphoprotein phosphatase 2A. The data represent the mean ± standard error of three biological replicates. Significant differences between the mean values are indicated (*** *p* ≤ 0.001, ** *p* ≤ 0.005, * *p* ≤ 0.05) (*U*-test).

Genes from the first group were partially overlapped with “response to abscisic acid stimulus”, i.e., the five genes *HVA22E*, *ABI4*, *ABI5*, *LTI65,* and *LTP4.* These genes were represented well among mono- and dicotyledonous plant species (Figures S5–S9). Among these genes, *ABI4*, *ABI5*, and *HVA22E* are highly conservative in functional domains (Tables S10 and S11). *LTI65* and *LTP4* are less conservative; their homologs have been identified only in the genomes of higher plants (Figures S8 and S9). Therefore, the essential gene *ABI3 (ABA INSENSITIVE 3)*-encoded AP2/B3-like transcriptional factor family protein (Psat3g142040) was selected from the second group for the qRT-PCR analysis. According to the analysis results, the expression of all genes corresponded to the predicted and significant decrease during the seed-to-seedling transition (Figure 7).

We obtained exciting results for three copies of genes represented in the *P. sativum* genome. The expression of Psat4g157880, Psat4g157760, and Psat3g105280 annotated as *LEA14* has differed between copies. The decrease in expression during the switching of the developmental stage was detected only for the gene Psat4g157880 (Figure 7). We did not make the qRT-PCR analysis of other genes. According to the RNA-seq results, the Psat4g157760 expression was close to zero, and Psat3g105280 expression was equally high before and after radicle protrusion. However, we obtained the opposite results for the *RD22* genes Psat6g033920, Psat6g033960, and Psat0s4403g0040. The expression level of all copies significantly decreased after radicle protrusion. These genes were designated as *RD22-1*, *RD22-1,* and *RD22-1*, respectively (Figure 7).

## 3. Discussion

Seed germination is considered the first developmental phase in higher plants’ life cycle, followed by post-germination growth and seedling development [2,49,50]. Until now, most studies focused on understanding the mechanisms of *sensu stricto* germination, but little is known about the molecular events taking place during the proper transition to the post-germination stage. Silva et al. analyzed the dynamics of the primary metabolites [50] and transcript profiles [51] in *A. thaliana* from the seed germination to seedling establishment. They revealed the two major metabolic shifts with changes in amino acids, organic acids, carbohydrates, and their derivatives. The first one occurred from the imbibition of dry seed to the early-seedling stages, including testa rupture, radicle protrusion, and root hair formation. The second occurred between radicle protrusion and seedling establishment when cotyledons fully opened. Correlation analysis between metabolite and transcript profile enabled the identification of the genes that influence the fluxes of metabolites during the seed-to-seedling transition.

In the present study, we focused on the seed-to-seedling transition in *Pisum sativum* L. Our experimental model included pea seeds after 72 h of germination sorted into two seed batches—just before and after the start of embryonic root growth (Figure 1). Radicle protrusion was accompanied by ROS generation in seed axes. The process of ROS accumulation during seed germination and the beneficial role of ROS in the regulation of cell elongation and division is well established [52]. We observed the increase in TBA-reactive products and hydrogen peroxide with localization of ROS in embryonic root and plumule (Figure 1 and Figure 2b,c). Since the germination process is accompanied by an increase in water content from the dry state at the beginning to complete tissue hydration at the end, we compared the moisture content in isolated seed axes. It was 72% and 87% before and after radicle protrusion, respectively (Figure 2a). This shift in the water status of the analyzed embryonic axes correlated with their desiccation tolerance. The seeds sorted before radicle protrusion survived after drying and developed normal seedlings, but those selected after radicle protrusion lost their desiccation tolerance and died after drying (Figure 2e).

The phenomenon of desiccation-tolerance acquisition in orthodox seeds is well-known in literature [11,27,28,29]. Its molecular basis is grounded on the accumulation during late maturation of such compounds as the LEA proteins, small heat shock proteins, non-reducing oligosaccharides of the raffinose group [11,53], glutathione [54], tocochomanols [55], and carotenoids [56].

Progressive water loss in matured seeds induces the so-called cellular “glassy state” and a slowdown of chemical reactions [57,58]. Such glassy matrix consists of soluble sugars, which immobilize macromolecules offering protection to membranes and proteins [10,53]. The viscosity of cytoplasm also increases, while water and oxygen diffusion are suppressed, and the rates of all chemical reactions become dramatically reduced [57,58,59]. Due to these shifts in the physicochemical properties of multiple cellular structures, orthodox seeds can maintain viability for decades [60]. However, there is little information about the molecular mechanism of desiccation-tolerance loss at the post-germination stage.

### 3.1. Key Genes of the Response to Drought Tolerance of Pea Seeds

We compared RNA-sequencing-based transcriptomics in the isolated embryonic axes before and after radicle protrusion and identified 24,184 DEGs with FDR < 0.05 and (|logFC|) > 2. The PCA analysis demonstrated a high level of differences between versions, with 99% of the explained information (Figure 3). After radicle protrusion, the expression of 2101 genes increased, and that of 415 genes decreased more than four times (Figure 4). Among the downregulated DEGs, the largest group included the GO terms “response to heat”, “response to abiotic stimulus”, “response to temperature stimulus”, “response to ROS”, “response to water”, and “response to water deprivation” (Figure S3a and Figure 5). At the same time, we observed the upregulation of the DEGs from the GO terms “response to stimuli”, “immune system processes”, and “immune effector process” (Figure S3b).

Annotation of the metabolic pathways using the MapMan software confirmed the significant genetic rearrangement of the general metabolic processes. Transition to the post-germination stage was accompanied by the gene expression related to photosynthesis, metabolism of lipids, sugars and amino acids, biosynthesis of cell wall components, redox status, and secondary metabolism (Figure 6a). The expression of the genes encoding heat shock proteins was mainly downregulated (Figure 6a). However, the expression of genes responsible for the ascorbate, glutathione, thioredoxin, and peroxidases level was increased (Figure 6b). Remarkably, we observed strong activation of the genes involved in defense responses to the biotic stress caused by fungi, bacteria, and viruses. These included the genes associated with pathogenesis-related (PR) proteins, salicylate- and JA-dependent defense pathways, and synthesis of terpenes, flavonoids, and phenylpropanoids.

The bioinformatic processing emphasized the genes from GO terms “response to water deprivation” and “response to abscisic acid stimulus” downregulated in seed axes after radicle protrusion. As is known, ABA is a phytohormone regulating the maintenance of seed dormancy and seed response to stress, particularly water deprivation [61]. Three genes (*LTI65*, *LTP4,* and *HVA22E*) were related to “response to water deprivation” and “response to abscisic acid stimulus” (Figure 7).

*LTI65/RD29B (LOW-TEMPERATURE-INDUCED 65/RESPONSIVE TO DESICCATION 29B)* encodes a protein expressed in response to such water deprivation conditions as cold, high-salt, and desiccation [62,63,64]. In the *Arabidopsis thaliana* genome, the promoter region of these gene contains two ABA-responsive elements (ABREs) [65]. In the genome of *P. sativum*, this gene encodes a protein with a total length of 1036 amino acids (aa) (Table S9). Analysis of phylogenetic similarity of *LTI65* based on full protein sequences showed no annotated homologues in the genomes of *Hordeum vulgare* L., some mosses, and drought-tolerant plants, such as the model resurrection specie *Selaginella moellendorffii* Hieron (Table S9, Figure S8).

*LTP4* encodes the lipid transfer protein, which is strongly up-regulated by ABA [66]. LPTs proteins can bind and transfer fatty acids, acylCoA esters, and several phospholipids [67]. *LTP4* acts redundantly with its closest homologue LTP3 by modulating the ABA pathway [66]. In *P. sativum*, *LTP4* encodes the probable lipid transfer protein (88aa). However, we did not find the *LTP4* homologues in the genomes of mosses and resurrection plants (Table S10, Figure S9). This family’s proteins are a members of PR-14 pathogenesis-related protein family [68]. Several plant LTPs act positively in plant disease resistance. *LTP3*, a pathogen- and ABA-induced gene, negatively regulates plant immunity in *A. thaliana*. *LTP3/LTP4* overexpression enhanced the susceptibility to virulent bacteria and compromised the resistance to avirulent bacteria [66]. LTP3 and its closest paralogue, *LTP4*, are highly induced during wilt disease development caused by the soil-borne pathogen *Ralstonia solanacearum* [69].

*HVA22* encodes one more of the ABA-induced LEA proteins isolated from the aleurone tissue of barley [70]. This protein is short (147aa) and highly conservative (Table S10, Figure S7). Previously the bioinformatic analysis has revealed that 354 HVA22 homologs are present in diverse eukaryotic organisms. These homologs share high amino acid sequence similarity in a conserved TB2/DP1 domain. HVA22E encodes one of five HVA22 homologs in *A. thaliana* and is upregulated in response to cold and salt stress, ABA treatment, or dehydration [71,72].

The three genes included only in the “response to water deprivation” group were *LEA14*, *RD22*, and *PER1*. Most *LEA* genes are known to have ABA response elements (ABRE) in their promoters, and their expression can be induced by ABA, cold, or drought [65,73]. The desiccation-related protein LEA14 belongs to Group II LEA proteins, also known as dehydrins [73,74,75]. *LEA14* was induced in response to salt and light stress in *A. thaliana* [76,77] and low temperatures in *Pyrus communis* L. [78].

*RD22* encodes proteins of the BURP family, members of which share a highly conserved BURP domain [79,80]. AtRD22 is up-regulated by high-salt, desiccation, and exogenously supplied ABA [81,82,83]. It is possible that RD22 also regulates cell wall peroxidases under stress conditions [84].

Peroxiredoxins (Prx) are thiol-dependent antioxidants containing one (1-Cys) or two (2-Cys) conserved Cys residues [85]. *PER1* encodes a protein 1-Cys peroxiredoxin. 1-Cys Prx (also named PER1) is expressed in developing seeds but disappears rapidly after germination [86,87]. PER1 has a vital function during the stages accompanied by dramatic ROS generation by different processes such as desiccation during late maturation [88], “imbibing stress”, and germination under abiotic stress [87,89]. 1-Cys Prxs function not only to relieve mild oxidative stresses but also as molecular chaperones under severe conditions during seed germination and plant development [90]. Overoxidation controls the switch in function of 1-Cys-Prxs from peroxidases to molecular chaperones [90]. Chen et al. (2020) demonstrated that AtPER1 eliminates ROS to suppress ABA catabolism and GA biosynthesis, thus improving the primary seed dormancy and making the seeds less sensitive to adverse environmental conditions [91].

Altogether, our data showed that after radicle protrusion in pea seed axes, there was a significant change in the pattern of genes responsible for adaptation to abiotic and biotic stresses. The seed and seedling demonstrated different pictures of gene expression in response to abiotic stresses. Seedlings lost their capability to survive after drying but acquired a system of defense against biotic stresses caused by fungi, bacteria, and viruses. Such massive rearrangements of gene expression programs should be under hormone control. We observed that genes related to hormone signaling changed differently (Figure 6b). Some of the ABA-related genes were upregulated, and some were downregulated. However, most genes encoding other hormones, such as auxin, ethylene, SA, and JA, were expressed.

### 3.2. The LAFL Network and the Developmental Program

As known, the master negative regulators of seed germination include the transcription factors LEC1, ABI3, FUS3, LEC2 (so-called LAFL), and DOG1 [16,41,92]. Switching the developmental program from maturation to germination is accompanied by gene suppression of the LAFL network and activation of the genes involved in vegetative growth [15]. Our results evidenced that among the *LAFL* genes, only *ABI3* was expressed in pea seed axes before radicle protrusion (Figure 7). The expression of *LEC1*, *LEC2*, *FUS3*, and *DOG1* was apparently suppressed at the early germination stage. Together with *ABI3*, we observed the expression of the ABA-dependent genes *ABI4* and *ABI5*.

*ABI3*, *ABI4,* and *ABI5* are the core transcriptional factors containing B3, AP2, and bZIP domains [93,94]. They control the expression of the ABA-responsible genes involved in the seed-specific events, such as maturation, dormancy, longevity, germination, and post-germination growth [61,95,96,97,98,99,100]. Interestingly, these genes are tightly co-expressed during all steps of plant ontogenesis [101]. *ABI3* and *ABI5* are involved in the regulation of chlorophyll catabolism, raffinose family oligosaccharides synthesis, and *LEA* genes expression at the late maturation stage [16,92,102,103,104,105]. *ABI5* can also act as a signaling integrator of ABA and other hormones [103,106,107]. Disruption of *ABI5* decreases the expression of many ABA-responsive genes, whereas *ABI5*-overexpressing plants were hypersensitive to ABA during seed germination and early seedling development [108]. *ABI4* is involved in hormone synthesis and signaling, seedling establishment, plastid-to-nucleus signaling, synthesis and breakdown of lipids, and disease resistance [109]. It was suggested that *ABI4* could be activated in response to redox signals involved in developmental processes and response to oxidative stress [110]. In seeds, *ABI4* is mainly expressed during late maturation and early germination, mediating the balance between ABA and GA and thereby regulating the dormancy [11,98,101,111].

It is interesting that *ABI4* and *ABI5* are the highly conservative genes in the diverse drought-tolerant species. *ABI5* is the only gene represented in genomes of resurrection plants *Xerophyta humilis* (Baker) T. Durand & Schinz and *S. moellendorffii* (Table S10, Figures S5 and S6). In *P. sativum* genome, they encode the proteins with the length of 240aa and 422aa, respectively.

In our study, the *ABI3*, *ABI4,* and *ABI5* genes were expressed before radicle protrusion but reduced to zero after the protrusion. Thus, the seed-to-seedling transition was associated with blocking the expression of the ABA-related genes *ABI3*, *ABI4*, *ABI5,* and *LEA14*, of which only *ABI3* belongs to the LAFL regulatory network. Together, we observed a sharp decrease in the expression of *HVA22E*, *LTI65/RD29B*, and *LTP4* involved in responses to water deprivation and *PER1* involved in the suppression of ABA catabolism and GA biosynthesis via ROS elimination.

## 4. Conclusions

Thus, this study provided a primary global view of transcriptomic changes during the seed-to-seedling transition in *P. sativum*. Seedling establishment was accompanied by growth initiation of embryonic roots, increased water content, and ROS accumulation. Importantly, seeds were tolerant to desiccation before radicle protrusion and survived after drying to initial moisture content. However, radicle protrusion resulted in the loss of desiccation tolerance, and seedlings died after drying. Through the transcriptomic comparison of embryonic axes isolated from 72-hour-germinated pea seeds before and after radicle protrusion, we deciphered the significant genetic rearrangement of metabolic processes. This rearrangement includes the upregulation of gene expression related to the photosynthesis; metabolism of lipids, sugars, and amino acids; and cell wall biosynthesis. It was accompanied by (i) a desiccation tolerance loss and (ii) the initiation of secondary metabolism and activation of genes involved in defense responses to biotic stress. Most genes associated with auxin, ethylene, salicylic, and jasmonic acids were upregulated. However, ABA-related genes demonstrated a different behavior. Several of them were upregulated, while others were downregulated. Among them, *ABI3*, *ABI4*, *ABI5*, and *LEA14* were expressed in embryonic axes before radicle protrusion but downregulated at the post-germination stage. Surprisingly, among the *LAFL* genes, only *ABI3* was expressed in pea seed axes before radicle protrusion. In summary, we observed the downregulation of ABA-related genes *HVA22E*, *LTI65/RD29B*, and *LTP4* were involved in responses to water deprivation and *PER1* involved in the suppression of ABA catabolism and GA biosynthesis via ROS elimination. These results provide the next step toward identifying the master regulators controlling seed-to-seedling transition in plants and open new perspectives for understanding the complex regulatory mechanisms underlying the seedling establishment. Further exploration of genes controlling the switch of stress-tolerance programs can facilitate the breeding of plants more tolerant to drought.

## 5. Materials and Methods

### 5.1. Preparation of Plant Material

Pea seeds of the commercial cultivar “Prima” were obtained from the N.I. Vavilov All-Russian Institute of Plant Genetic Resources (St. Petersburg, Russia). Seeds were incubated between layers of moist filter paper. Seeds imbibed for 72 h were visually divided into two batches: (a) before the start of embryonic root growth (before radicle protrusion) and (b) after the start of root growth (after protrusion). Seed axes from both batches were isolated, frozen in liquid nitrogen, and stored at −80 °C before DNA and RNA extraction.

### 5.2. Physiological Evaluation

*In situ localization of the superoxide anion.* Pea seeds germinated for 72 h, before and after radicle protrusion, were incubated in 6 mM nitroblue tetrazolium (NBT) in 10 mM Tris-HCl buffer (pH 7.4) at room temperature for 30 min [112]. The superoxide anion was visualized as deposits of dark-blue insoluble formazan compounds [113].

*TBA-reactive substances*. Content of thiobarbituric acid (TBA)-reactive substances were quantified as malondialdehyde equivalents (MDA) as described by [114]. TBA content was measured separately using five replicates. The differences among variants were tested with Past software (https://www.nhm.uio.no/english/research/infrastructure/past accessed on 18 May 2022), taking *p* ≤ 0.05 as significant.

*Hydrogen peroxide content*. The hydroperoxide content was calculated as 13S-hydroperoxy-9*Z*, 11*E*-octadecanoic acid equivalents [115] with some modifications described by [116]. Hydrogen peroxide content was measured separately using nine replicates. The differences among variants were tested with Past software (https://www.nhm.uio.no/english/research/infrastructure/past, accessed on 18 May 2022), taking *p* ≤ 0.001 as significant.

*Seed moisture content.* Moisture content was measured separately in seed axes and cotyledons using three replicates of 9 seeds each by oven method at 103 °C for 17 h. The calculation was done on a dry weight basis, with the degree of moisture expressed as a percentage. The differences among variants were tested with Past software (https://www.nhm.uio.no/english/research/infrastructure/past, accessed on 18 May 2022), taking *p* ≤ 0.05 as significant.

*Desiccation tolerance*. Seed desiccation tolerance was assessed based on the embryonic axes survival after drying. We incubated seeds between layers of moist filter paper at 22 °C. After 24, 48, and 72 h of germination, we transferred the seeds to dry paper and dried them to the initial moisture for three days at 22 °C. Dried seeds were returned to moist filter paper to continue the germination process.

*Seed germination*. Twenty-five seeds in 4 replicates were incubated between layers of moist filter paper at 22 °C for nine days. The number of seeds with visible radicle protrusion was counted for each day. After nine days, the numbers of normally developed seedlings, abnormally developed seedlings, and non-germinated seeds were calculated [117]. The differences among variants were tested with Past software (https://www.nhm.uio.no/english/research/infrastructure/past, accessed on 18 May 2022), taking *p* ≤ 0.05 as significant.

### 5.3. Isolation of Total DNA and RNA

The seed axes were frozen in liquid nitrogen and homogenized with a pestle in a mortar. According to the manufacturer’s instructions, total DNA was isolated using the DNeasy Plant Mini Kit (QIAGEN, Hilden, Germany) (www.qiagen.com, accessed on 18 May 2022). Total RNA was isolated using the RNeasy Plant Mini Kit (QIAGEN) according to the manufacturer’s instructions (www.qiagen.com, accessed on 18 May 2022), followed by DNase treatment with the RNase-free DNase set (QIAGEN). The quality of the isolated DNA and RNA was evaluated in 1% agarose gel prepared based on the TAE buffer with the addition of ethidium bromide as an intercalating dye. The Sky-High 250 b–10 kb marker (BioLabMix, Novosibirsk, Russia) was used as a DNA molecular weight marker. The resulting isolation products were visualized using the BioRad ChemiDoc MP gel-documenting system (Bio-Rad Laboratories, Moscow, Russia). Concentrations of the isolated DNA and RNA were measured using a NanoDrop™ 2000/2000c spectrophotometer (Thermo Fisher Scientific Inc., Waltham, MA, USA).

### 5.4. RNA Library Preparation

The TruSeq mRNA-stranded reagent kit (Illumina, San Diego, CA, USA) was employed to enrich total RNA samples with the poly(A+) fraction, followed by cDNA synthesis using Superscript II Reverse Transcriptase, followed by the second strand cDNA synthesis. cDNA was used to prepare libraries compatible with Illumina sequencing technology. The quality of the obtained libraries was checked using the Fragment Analyzer (Agilent, Moscow, Russia). Quantitative analysis was performed by real-time polymerase chain reaction (qPCR). After quality control and DNA quantity evaluation, the library pool was sequenced using the Illumina NovaSeq 6000 SP high-throughput whole-genome sequencing system (www.illumina.com, accessed on 18 May 2022).

### 5.5. Reads Preprocessing, Mapping and Differential Expression Analysis

The quality of raw reads was checked using FASTQC v. 0.11.9 [118]. Filtering of the libraries was performed using the bbmap software (https://sourceforge.net/projects/bbmap/, accessed on 12 March 2021). It resulted in removing unidentified bases (N) or bases with a Phred quality score below 20 from both 3′- and 5′-ends of the read and removing the reads with a length less than 60. The filtered reads were then mapped to the Pea Genome Assembly v1a from the UGRI server (https://urgi.versailles.inra.fr/Species/Pisum, accessed on 12 March 2021). The Hisat2 tool was applied to map the short-read libraries to the reference genome [119]. The number of reads aligned to each gene was counted with the Subread software package [120]. The PCA analysis using DESeq2 was conducted to assess library quality and search for outliers [121]. The differential gene expression analysis was performed using the edgeR package for R [122]. Genes with low expression were eliminated. Genes with a false-discovery rate (FDR) < 0.05 and log base 2-transformed fold change (|logFC|) > 2 were considered differentially expressed. A volcano plot showing the distribution of differentially expressed genes (DEGs) was made using the EnhancedVolcano package [123].

### 5.6. Differentially Expressed Genes (DEGs) Annotation

DEGs were annotated with the blastx search against the *A. thaliana* (TAIR 10) protein database (with a threshold e-value < 10 × 10^−9^). The GO and MapMan annotations were assigned based on the *A. thaliana* homologous proteins. The AgriGO v.2 toolkit was used to perform singular enrichment analysis of DEG lists [124]. The GO terms with an adjusted *p*-value < 0.05 were considered significantly enriched. Reduction of redundancy and TreeMap visualization of GO terms were performed using the ReviGO tool [125]. The diversity and abundance of differentially expressed genes were investigated with MapMan using the *A. thaliana* mapping database (Ath_AGI_ISOFORM_MODEL_TAIR10_Aug2012) downloaded from the MapMan server [126]. The full list of DEGs (upregulated and downregulated) was used as a query experiment and mapped on the metabolism overview and stress metabolism pathways.

### 5.7. Quantitative Reverse-Transcription PCR (qRT-PCR) and Sequence Analysis

A single-stranded cDNA was synthesized from the RNA template using the M-MuLV–RH First Strand cDNA Synthesis Kit (BioLabMix, Novosibirsk). Quantitative PCR was performed using the SYNTOL SYBR Green I+ROX kit (Syntol) on the CFX96 Touch Real-Time PCR Detection System (BioRad). PCR was made in 15 μL of the reaction mixture under the following conditions: 1 cycle at 50 °C for 10 min; 1 cycle at 95 °C for 5 min; 40 cycles at 95 °C for 10 s and 60 °C for 30 s. PCR product melting curves were constructed under the following conditions: 10 s at 95 °C; 5 s at 65 °C; and 5 s at 95 °C. To standardize the amount of cDNA template, qPCR was performed with the primers for the housekeeping gene *Phosphoprotein Phosphatase 2a* (GenBank: Z25888): 5′-ccacattacctgtatcggatgaca-3′ and 5′-gagcccagaacaggagctaaca-3′ [127]. Each sample was amplified in three technical replicates. Plots of the dependence of the threshold cycle on the initial concentration of matrices were built based on three consecutive 3-fold dilutions. The differences among genotypes were tested by *U*-test in Past software (https://www.nhm.uio.no/english/research/infrastructure/past, accessed on 18 May 2022), taking *p* ≤ 0.001, *p* ≤ 0.005, and *p* ≤ 0.05 as significant. The primer design for determining the relative level of gene expression was made using the IDT PrimerQuest software (http://eu.idtdna.com/PrimerQuest/Home, accessed on 19 June 2021) and Oligo Primer Analysis Software v.7 (https://www.oligo.net accessed on 19 June 2021) (Table S9). The annotation of the functional domains was carried out using MOTIF Search (https://www.genome.jp/tools/motif/, accessed on 19 June 2021). The evolutionary analysis was performed in MEGA X software (http://www.megasoftware.net, accessed on 19 June 2021) [128] by maximum likelihood method and JTT matrix-based model [129] with 1000 bootstrap replicates. LALIGN/PLALIGN has been used to identify percent sequence identity (https://fasta.bioch.virginia.edu/fasta_www2/fasta_www.cgi?rm=lalign&pgm=lal, accessed on 19 June 2021).

## Supplementary Materials

The following supporting information can be downloaded at: https://www.mdpi.com/article/10.3390/plants11131686/s1, Figure S1: Appearance of the embryonic axes of normally and abnormally developed 9-day-old seedlings; Figure S2: Appearance of 14-day-old; Figure S3: Treemap visualization of GO terms significantly enriched in genes downregulated and upregulated in pea seed axes after seed radicle protrusion; Figure S4: qRT-PCR validation of RNA-seq results; Figure S5: Analysis of phylogenetic similarity of ABI4 genes based on full protein sequences; Figure S6: Analysis of phylogenetic similarity of ABI5 genes based on full protein sequences; Figure S7: Analysis of phylogenetic similarity of HVA22E genes based on full protein sequences; Figure S8. Analysis of phylogenetic similarity of LTI65 genes based on full protein sequences; Figure S9: Analysis of phylogenetic similarity of LTP4 genes based on full protein sequences; Table S1: Statistics of the obtained RNA-seq libraries; Table S2: Raw counts; Table S3: RPKMs; Table S4: Differential expression of *Pisum* genes in seed axes after radicle protrusion; Table S5: Result of the BLASTX search of *P. sativum*. DEGs against *A. thaliana* protein database (TAIR10); Table S6: GO term; Table S7: Genes mapped to the overview of the metabolic pathway using MapMan; Table S8: Genes mapped to the stress metabolic pathway using MapMan; Table S9: Primers used in this work; Table S10: The percentage of identity of proteins of *P. sativum* and other plant species; Table S11: Functional domains of *P. sativum* proteins.
