# Supplementary material for: Seed-to-Seedling Transition in Pisum sativum L.: A Transcriptomic Approach
Source: Plants (Basel). 2022 Jun 25;11(13):1686. doi: 10.3390/plants11131686 (PMC9268910; doi:10.3390/plants11131686)
Supplement: Supplementary file 1 [file plants-11-01686-s001.zip › Figures S1-S9.pdf]

plants

MDPI

Supplements

Seed-to-Seedlings Transition in *Pisum sativum* L.: A Transcriptomic Approach

Galina Smolikova [1,*], Ksenia Strygina [1], Ekaterina Krylova [1,2], Aleksander Vikhorev [3], Tatjana Bilova [1], Andrej Frolov [3,4], Elena Khlestkina [2] and Sergei Medvedev [1]

[1] Department of Plant Physiology and Biochemistry, St. Petersburg State University, 199034 St. Petersburg, Russia; g.smolikova@spbu.ru (G.S.); t.bilova@spbu.ru (T.B.); k.strygina@spbu.ru (K.S.); s.medvedev@spbu.ru (S.M.)
[2] Postgenomic Studies Laboratory, Federal Research Center N.I. Vavilov All-Russian Institute of Plant Genetic Resources of Russian Academy of Sciences, 190000 St. Petersburg, Russia; e.krylova@vir.nw.ru (E.K.); khlest@bionet.nsc.ru (E.Kh.)
[3] Institute of Cytology and Genetics, Siberian Branch of Russian Academy of Sciences, 630090, Novosibirsk, Russia; vikhorev@bionet.nsc.ru (A.V.)
[3] Department of Biochemistry, St. Petersburg State University, 199034 St. Petersburg, Russia
[4] Department of Bioorganic Chemistry of Leibniz Institute of Plant Biochemistry, 06120 Halle (Saale), Germany; andrej.frolov@ipb-halle.de (A.F.)
* Correspondence: g.smolikova@spbu.ru

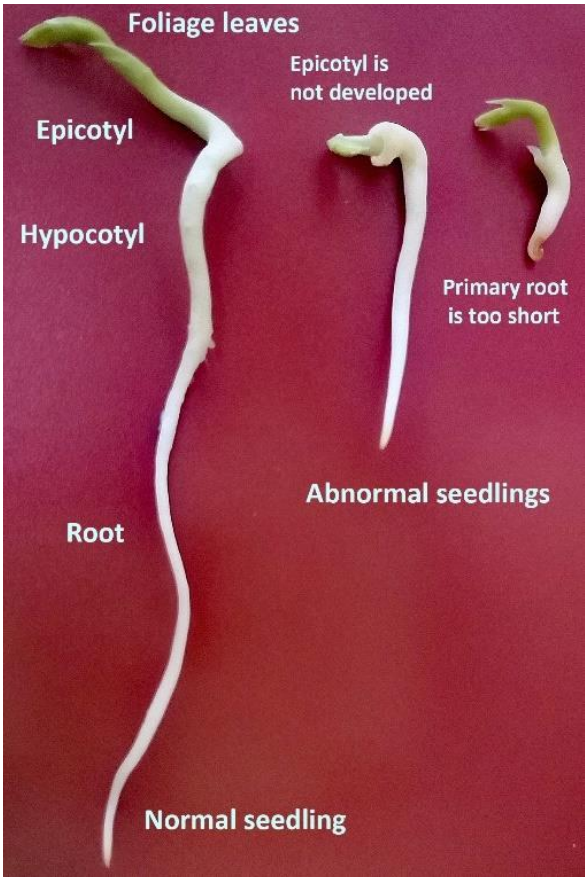

Figure S1. Appearance of the embryonic axes of normally developed and abnormally developed 9-day-old seedlings of pea cv. Prima. Axes include the root, hypocotyl, epicotyl and plumule with true leaves.

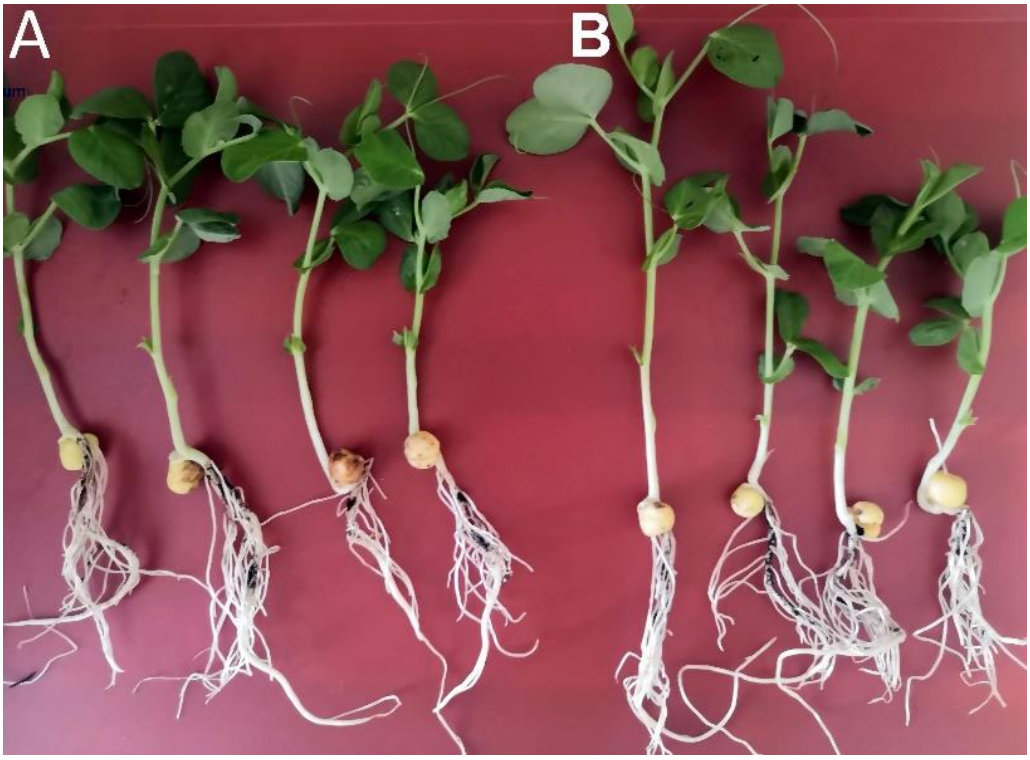

Figure S2. Appearance of 14-day-old seedlings of pea cv. Prima: A - untreated seeds (control), B – seeds were imbibed for 72 h, then dried to the initial moisture content.

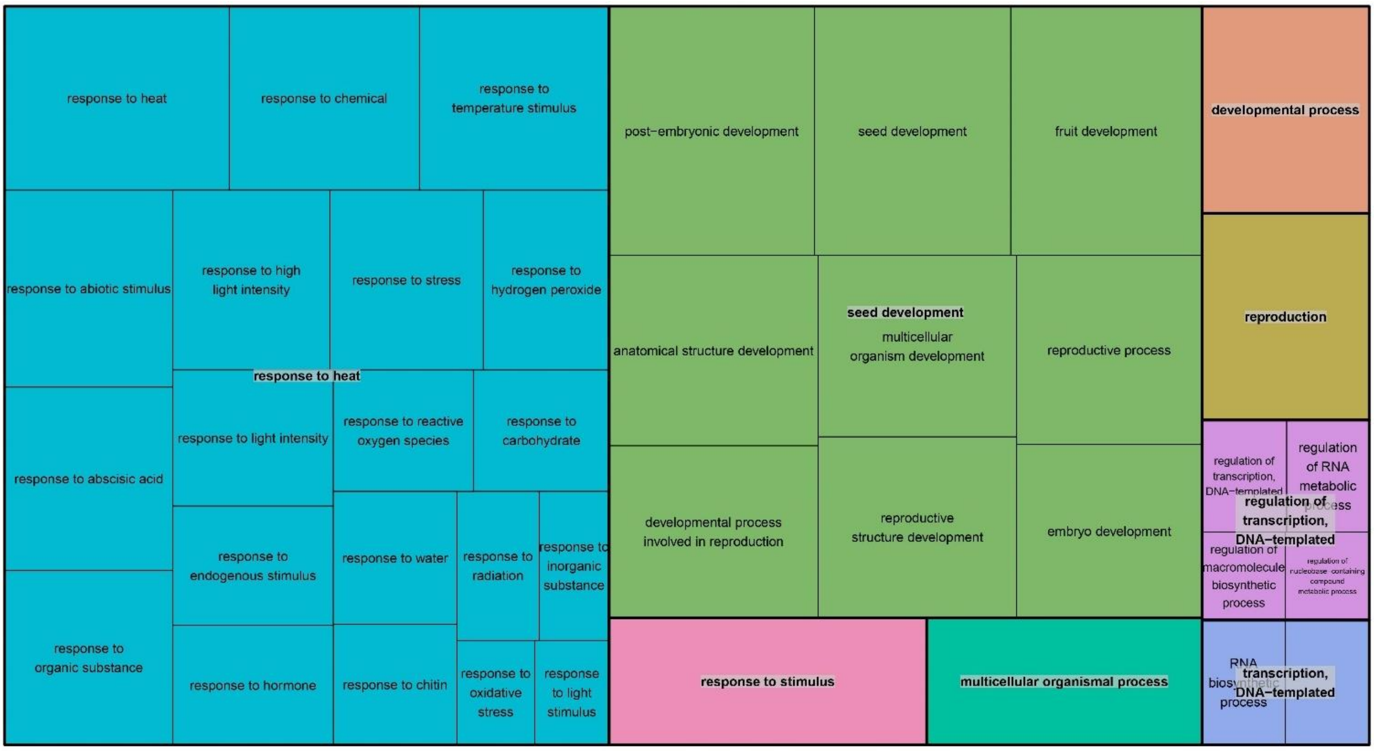

(**a**)

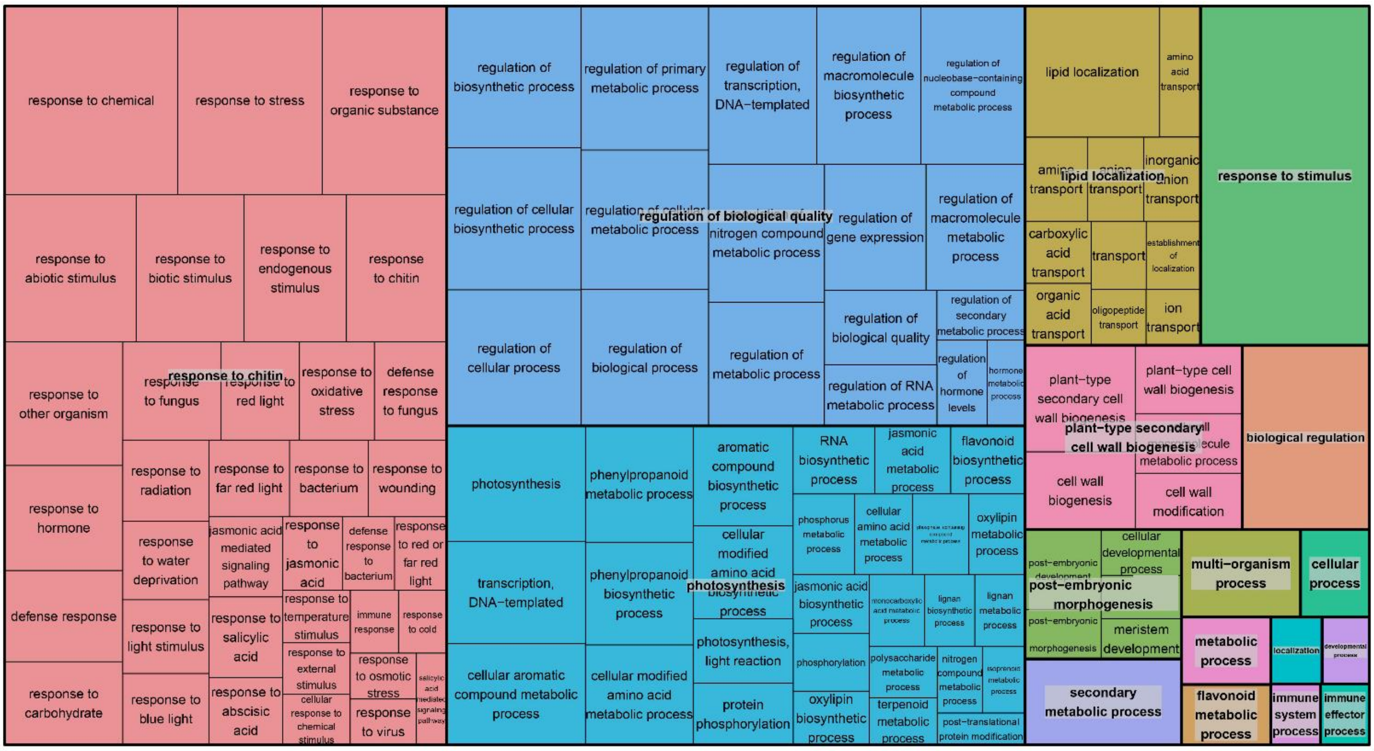

(**b**)

Figure S3. Treemap visualization of gene ontology (GO) terms significantly enriched in genes downregulated (**a**) and upregulated (**b**) in pea seed axes after seed radicle protrusion. Each rectangle corresponds to a GO term. The sizes of the rectangles reflect the *p*-value: the bigger is the size, the smaller is the *p*-value. The color indicates clusterization of GO terms by semantic similarity. The representative GO term of each cluster is shown in the center of a cluster on a grey background.

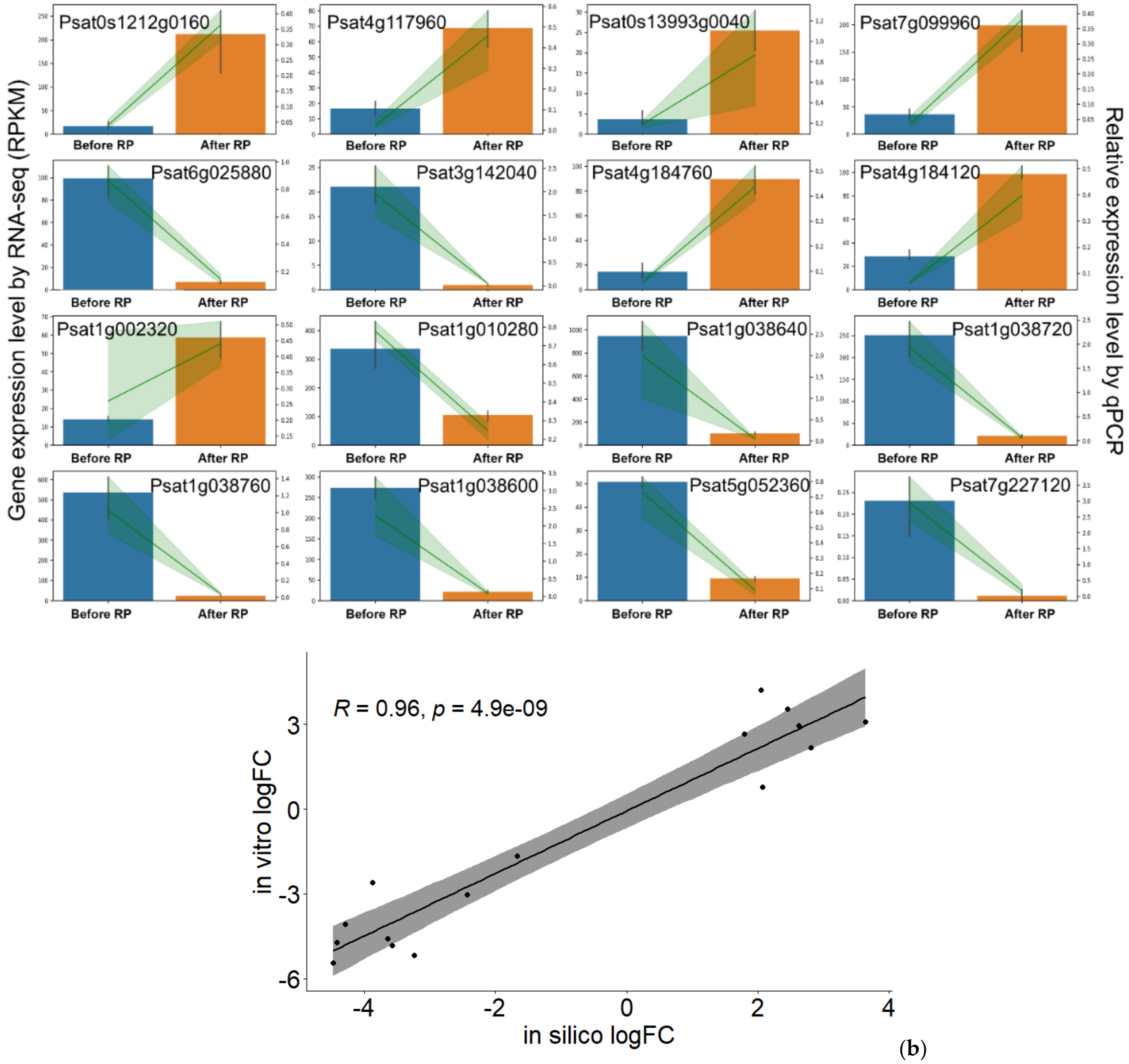

(**a**)

(**b**)

Figure S4. Real-time quantitative PCR (qRT-PCR) validation of RNA-seq results. (**a**) Expression of 16 up- and downregulated DEGs validated by qRT-PCR (*in vitro*) and compared with their expression obtained from RNA-Seq (*in silico*). Bars with standard errors represent the relative expression level determined by qRT-PCR (right Y-axis). Blue bars indicate the gene expression in seed axes before radicle protrusion (RP), and orange bars indicate the gene expression in seed axes after RP. Green lines with standard errors indicate transcript abundance based on RPKM values according to RNA-Seq (left Y-axis). (**b**) Scatterplot representing correlation between RNA-seq and qPCR expression data. The X-axis corresponds to the log2-transformed expression change obtained from RNA-seq data. The Y-axis corresponds to the log2-transformed expression change obtained from qPCR data. Scattered points represent each gene. Spearman's coefficient of correlation (R) and *p*-value are reported in the top of the graph.

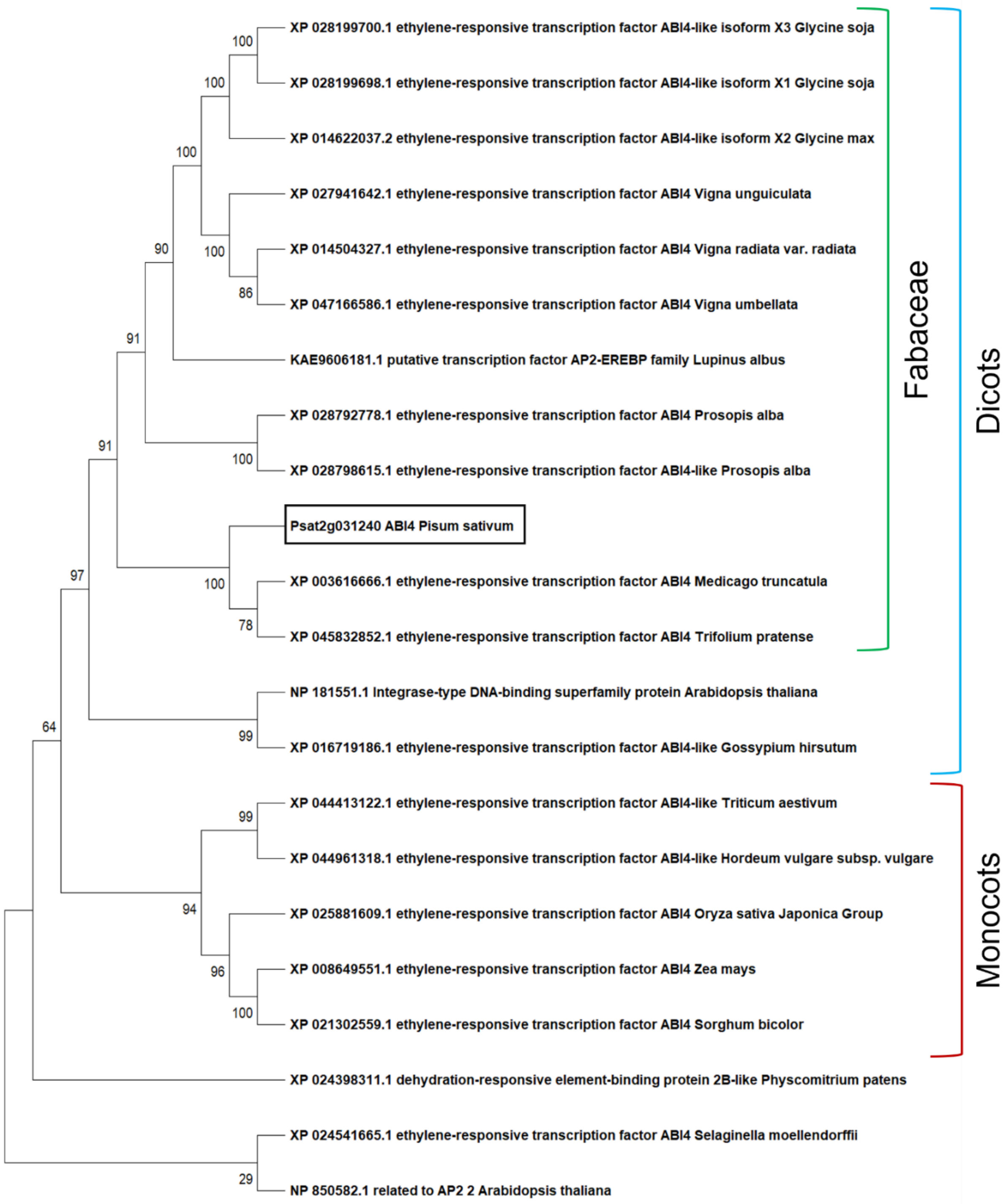

Figure S5. Analysis of phylogenetic similarity of ABI4 genes based on full protein sequences. The phylogenetic tree reconstruction was made in MEGA X. The Maximum likelihood method with 1000 bootstrap replicates was used for the phylogenic tree construction. The tree with the highest log likelihood (-10257.90) is shown. The percentage of trees in which the associated taxa clustered together is shown next to the branches. Initial tree(s) for the heuristic search were obtained automatically by applying Neighbor-Join and BioNJ algorithms to a matrix of pairwise distances estimated using the JTT model, and then selecting the topology with superior log likelihood value. This analysis involved 22 amino acid sequences. There were a total of 471 positions in the final dataset. The ABI4 protein from *P. sativum* is highlighted with a black rectangle. Fabaceae are indicated in green. Dicots are indicated in blue. Monocots are indicated in red.

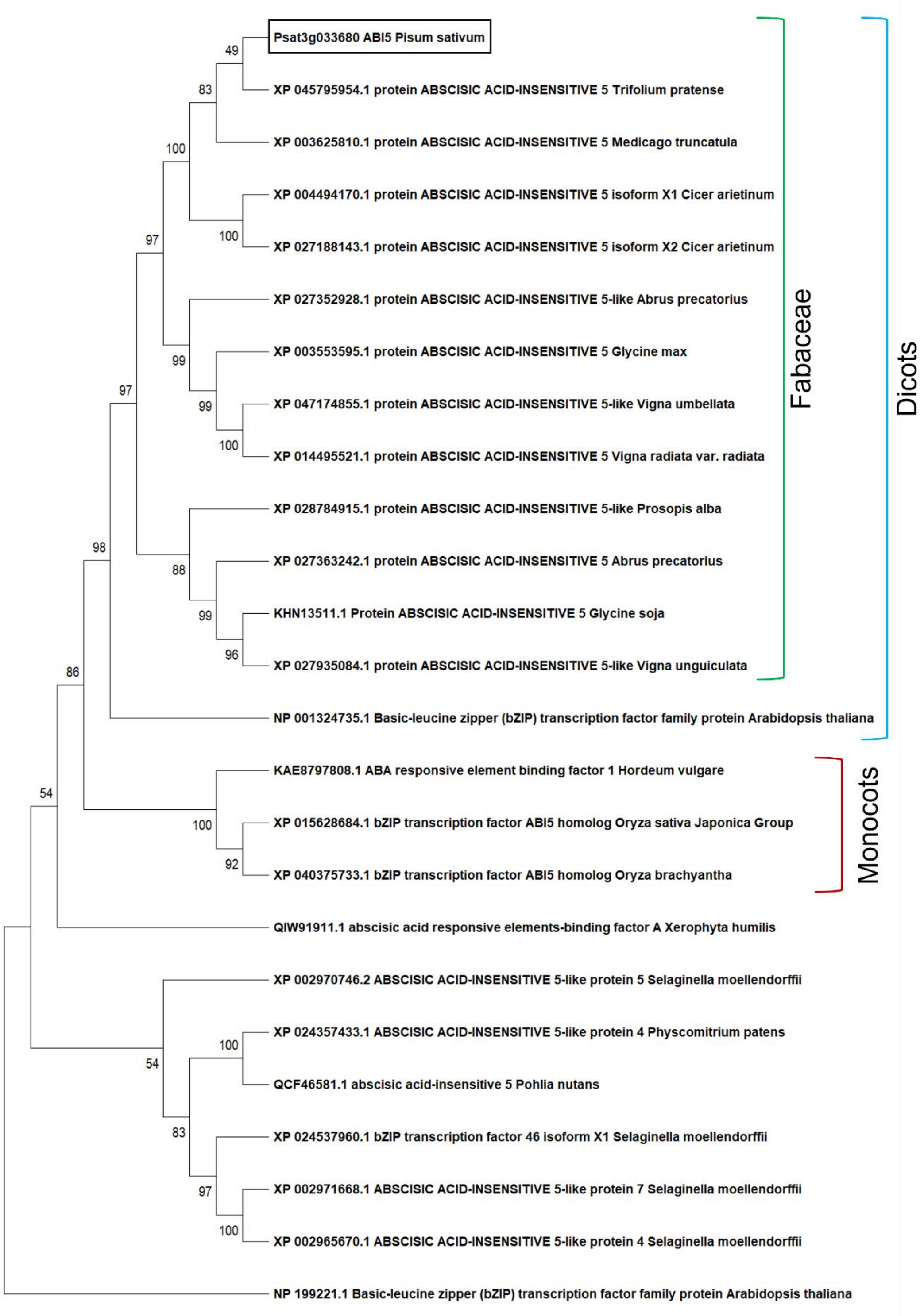

Figure S6. Analysis of phylogenetic similarity of ABI5 genes based on full protein sequences. The phylogenetic tree reconstruction was made in MEGA X. The Maximum likelihood method with 1000 bootstrap replicates was used for the phylogenic tree construction. The tree with the highest log likelihood (-13480.61) is shown. The percentage of trees in which the associated taxa clustered together is shown next to the branches. Initial tree(s) for the heuristic search were obtained automatically by applying Neighbor-Join and BioNJ algorithms to a matrix of pairwise distances estimated using the JTT model, and then selecting the topology with superior log likelihood value. This analysis involved 25 amino acid sequences. There were a total of 580 positions in the final dataset. The ABI5 protein from *P. sativum* is highlighted with a black rectangle. Fabaceae are indicated in green. Dicots are indicated in blue. Monocots are indicated in red.

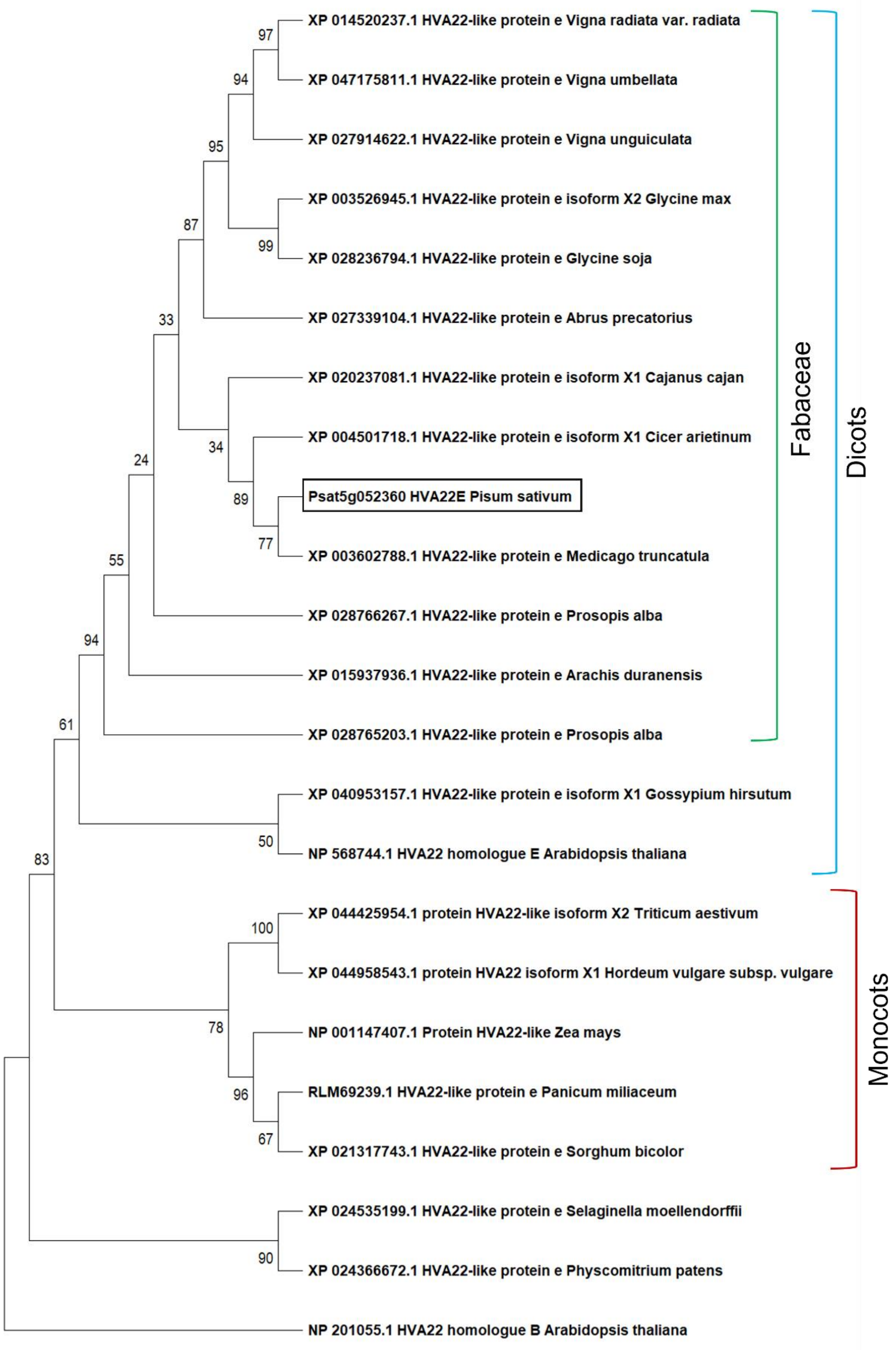

Figure S7. Analysis of phylogenetic similarity of HVA22E genes based on full protein sequences. The phylogenetic tree reconstruction was made in MEGA X. The Maximum likelihood method with 1000 bootstrap replicates was used for the phylogenic tree construction. The tree with the highest log likelihood (-3646.40) is shown. The percentage of trees in which the associated taxa clustered together is shown next to the branches. Initial tree(s) for the heuristic search were obtained automatically by applying Neighbor-Join and BioNJ algorithms to a matrix of pairwise distances estimated using the JTT model, and then selecting the topology with superior log likelihood value. This analysis involved 23 amino acid sequences. There were a total of 176 positions in the final dataset. The HVA22E protein from *P. sativum* is highlighted with a black rectangle. Fabaceae are indicated in green. Dicots are indicated in blue. Monocots are indicated in red.

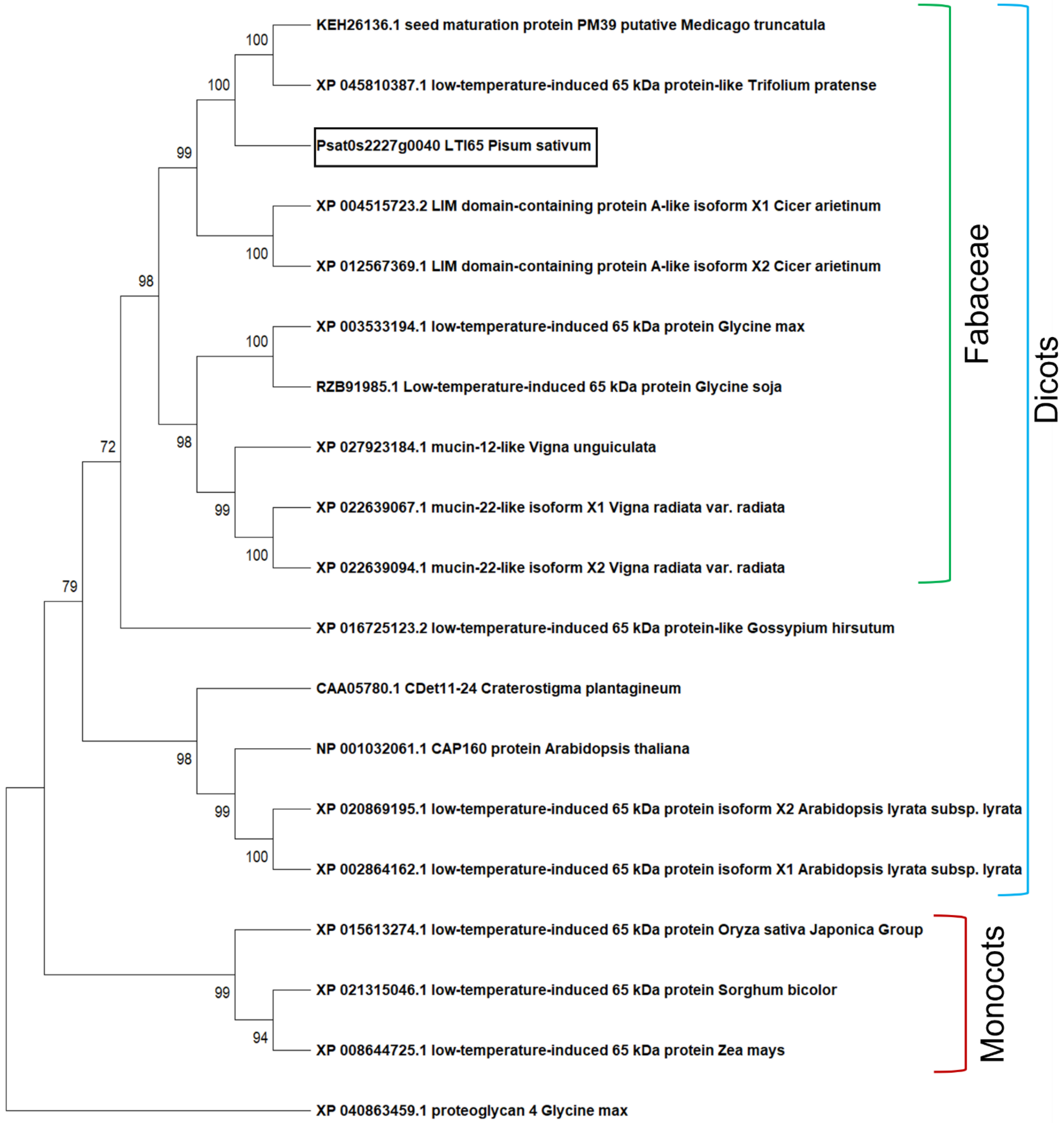

Figure S8. Analysis of phylogenetic similarity of LTI65 genes based on full protein sequences. The phylogenetic tree reconstruction was made in MEGA X. The Maximum likelihood method with 1000 bootstrap replicates was used for the phylogenic tree construction. The tree with the highest log likelihood (-21179.16) is shown. The percentage of trees in which the associated taxa clustered together is shown next to the branches. Initial tree(s) for the heuristic search were obtained automatically by applying Neighbor-Join and BioNJ algorithms to a matrix of pairwise distances estimated using the JTT model, and then selecting the topology with superior log likelihood value. This analysis involved 19 amino acid sequences. There were a total of 1753 positions in the final dataset. The LTI65 protein from *P. sativum* is highlighted with a black rectangle. Fabaceae are indicated in green. Dicots are indicated in blue. Monocots are indicated in red.

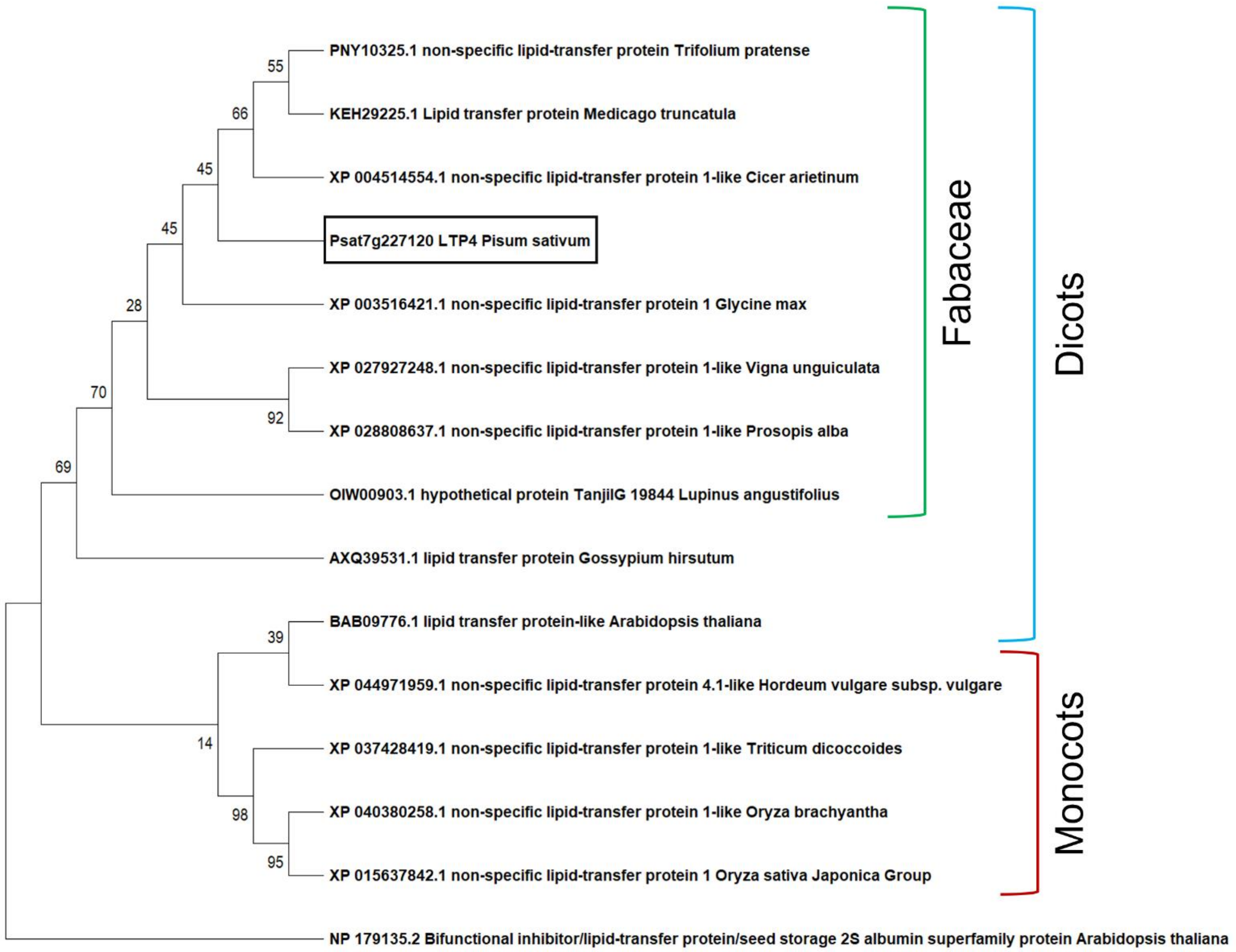

Figure S9. Analysis of phylogenetic similarity of LTP4 genes based on full protein sequences. The phylogenetic tree reconstruction was made in MEGA X. The Maximum likelihood method with 1000 bootstrap replicates was used for the phylogenic tree construction. The tree with the highest log likelihood (-2961.24) is shown. The percentage of trees in which the associated taxa clustered together is shown next to the branches. Initial tree(s) for the heuristic search were obtained automatically by applying Neighbor-Join and BioNJ algorithms to a matrix of pairwise distances estimated using the JTT model, and then selecting the topology with superior log likelihood value. This analysis involved 15 amino acid sequences. There were a total of 149 positions in the final dataset. The LTP4 protein from *P. sativum* is highlighted with a black rectangle. Fabaceae are indicated in green. Dicots are indicated in blue. Monocots are indicated in red.
